# Correlation between fibroblast growth factor receptor mutation, programmed death ligand-1 expression and survival in urinary bladder cancer based on real-world data

**DOI:** 10.3389/pore.2023.1611077

**Published:** 2023-04-21

**Authors:** Janos Revesz, Boglarka Posfai, Laszlo Pajor, Timea Papdan, Linda Varga, Viktor R. Paczona, Zoltan Varga, Farkas Sukosd, Aniko Maraz

**Affiliations:** ^1^ PhD School, University of Szeged, Szeged, Hungary; ^2^ Department of Oncotherapy, University of Szeged, Szeged, Hungary; ^3^ Department of Urology, University of Szeged, Szeged, Hungary; ^4^ Department of Pathology, University of Szeged, Szeged, Hungary

**Keywords:** urinary bladder cancer, fibroblast growth factor receptor, FGFR mutation, programmed death-ligand 1 expression, combined positive score

## Abstract

**Background:** Programmed cell death (PD)-1/PD-ligand 1 (PD-L1) inhibitors have made a breakthrough in the therapy of advanced urothelial bladder cancer (UBC). The impact of Fibroblast Growth Factor Receptor 3 (FGFR3) mutation on the effectiveness of PD-L1 treatment remains still unclear. Objective: Our study aimed to investigate the frequency of FGFR mutations at different tumor stages, and their relation to PD-L1 status and survival.

**Methods:** 310 patients with urothelial bladder cancer and subsequent radical cystectomy were included in a retrospective study over a 10-year study period at the University of Szeged, Hungary. FGFR3 mutations from the most infiltrative areas of the tumor were analyzed by targeted next-generation sequencing and PD-L1 (28-8 DAKO) tests (tumor positive score -TPS and combined positives score–CPS). In T0 cases FGFR3 mutations were analyzed from the earlier resection samples. Survival and oncological treatment data were collected from the National Health Insurance Fund (NHIF). Neoadjuvant, adjuvant and palliative conventional chemotherapies were allowed; immunotherapies were not. The relationship between the covariates was tested using chi-square tests, and survival analysis was performed using the Kaplan-Meier model and Cox proportional hazards regression.

**Results:** PD-L1 and FGFR could be tested successfully in 215 of the 310 UBC samples [pT0_cyst_ 19 (8.8%); St.0-I 43 (20%); St.II 41 (19%); St.III-IV 112 (52%)]. Significant pairwise dependency was found between tumor stage, FGFR3 mutation status and PD-L1 expression (*p* < 0.01). Samples with FGFR mutation were more common in less advanced stages and were also less likely to demonstrate PD-L1 expression. The effect of all investigated factors on survival was found to correlate with tumor stage.

**Conclusion:** FGFR alteration frequency varied between the different stages of cancer. Higher positivity rates were observed at early stages, but lower levels of PD-L1 expression were detected in patients with FGFR mutations across at all stages of the disease.

## Introduction

Bladder cancer is the tenth most common cancer worldwide with approximately 550,000 new cases annually (1). The depth of tumor invasion is the most important prognostic factor from a clinical standpoint and is divided into non-muscle-invasive bladder cancer (NMIBC) and the prognostically less favorable muscle-invasive cancer (MIBC) types (2). The rate of occurrence of MIBC capable of forming distant metastases is 25%–42%, while that of the disseminated stage is 4%–15% (1, 3). Localized MIBCs become disseminated in almost 50% over the course of the disease despite the radical cystectomy or locoregional trimodal therapy (3). In the treatment of advanced disease, for decades only combined chemotherapy was available, with relatively low efficacy and significant toxicity—moreover, molecular markers did not exist for predicting treatment ineffectiveness.

In recent years, checkpoint inhibitor immunotherapy has revolutionised the treatment of advanced urothelial bladder cancer (3). However, the role of potential biomarkers predicting the effectiveness of immunotherapy remains incompletely understood, and many factors that assume an immunogenic mechanism are currently under investigations.

In some studies, the presence of tumor infiltrating lymphocytes such as CD8^+^ (cluster of differentiation 8) T cells, as well as interferons and chemokines has been found to result in improved response to immunotherapies (4); however the prognostic value of programmed cell death ligand-1 (PD-L1) in urothelial cancer remains controversial (5). Based on several previous analyses, it can be assumed that patients with tumor cells showing PD-L1 positivity have a better response to anti-PD-1/PD-L1 monotherapy (6). The predictive effect of high PD-L1 expression on pembrolizumab immunotherapy has been confirmed in the first-line treatment of metastatic patients unfit for cisplatin (7, 8), as high combined positive score (CPS) of ≥10% was associated with a prolonged median overall survival (OS) (8).

In addition to immune mechanisms, the mutations responsible for bladder tumor progression are also the focus of genetic analyses. The mutation rate of urothelial carcinomas was published in The Cancer Genom Atlas (TCGA), however the possibilities and actual effectiveness of targeted drug treatments against mutations remain low (9). MIBC is a molecularly diverse disease with heterogeneous clinical outcomes (10, 11). Several reports have highlighted the clinical significance of molecular stratification of MIBC. A Consensus Molecular Classification of MIBC identified six different molecular classes with the occurrence of the following possible mutations: luminal papillary (24%)—FGFR3, KDM6A, STAG2; luminal non-specified (8%)—ELF3; luminal unstable (15%)—TP53, ERCC2, TMB+, APCBEC+; stroma-rich (15%), basal/squamous (35%)—EGFR+, TP53+, RB1+; and neuroendocrine-like (3%)—TP53-, RB1, by suggesting that responses to immunotherapy and chemotherapy may be enriched in specific subtypes (10). Because of the molecular heterogeneity of bladder cancer, molecular characterization is a very dynamically developing area.

In recent years, due to the emergence of FGFR inhibitor therapy, the clinical significance of FGFR mutation has come into view. Fibroblast growth factor receptor 3 (FGFR3) is a member of protein tyrosine kinase family, which consists of four transmembrane receptors, (FGFR1–4), and the alteration of the receptors induces an oncogenic signaling pathway (12). The aberrations in FGFR1–4—are detected in 5%–10% of all human cancers, although some types, such as urothelial cancer and intrahepatic cholangiocarcinoma display an increased (10%–30%) frequency of FGFR aberrations. Amongst these aberrations, the FGFR3 activating point mutation is the most frequently occurring one (10%–60%), mainly present in low grade, early stage NMIBC, while FGFR3 fusion and FGFR1 amplification can also occur in 6% and 7%, respectively (13).

However point mutation is rarely associated with MIBC, as nearly the half of advanced stage tumors bear wild-type FGFR3 gene (14). The FGFR pathway is an appealing targeted treatment option, and in the case of its alteration, phase 2 results of the multiple receptor inhibitor erdafitinib therapy are already available (15).

Sweis et al categorised bladder cancer into two subgroups using immune gene profiling; T-cell-inflamed tumors and non-T-cell-inflamed tumors. In the non-T-cell-inflamed subgroup, which is mostly associated with luminal-papillary subtype (or cluster I subtype), they identified some exclusively typical somatic mutation, where FGFR3 was the most common molecular alteration (16, 17). Lower response rates and shortened OS following anti–PD-L1 therapy was observed in patients with FGFR alterations (18).

Based on the published data, the ratio of PD-L1 expression, CPS score, and FGFR expression in each tumor stage is not clear, nor is the prognostic or predictive effect of their relation to each other.

The aim of our study was to demonstrate the frequency of FGFR3 mutation in different tumor stages of cystectomy samples, and to reveal a possible relationship between the FGFR status, PD-L1 status, CPS score, tumour-stages and the survival of patients.

## Material and methods

### Patients and demographic characterization

Prospective next-generation sequencing (NGS) of tumor tissues, and retrospective collections and analyses of clinical data were performed by the collaboration between University of Szeged, and the Szeged Biology Research Institute, with the use of Hungarian National Health Insurance Fund Database. Enrolled patients were previously diagnosed with urothelial bladder cancer and underwent radical operation during a 10-year period (before the immunotherapy era, between 2006 and 2016) at the University of Szeged, Hungary. Patients were included after partial or radical cystectomy, without known metastatic disease. The indication for the majority of cystectomies was primarily diagnosed muscle invasive transitional cell bladder cancer. In a smaller proportion of cases, extensive, multiple recurrent, non-muscle-invasive tumors were also indications for surgery, based on the guidelines. Neoadjuvant chemotherapy was allowed. The pT0 cases based on cystectomy specimens were called pT0_cyst_. In these cases the biomarker analysis was performed from the initial sampling tissues, but the stage was not redefined based on the less accurate result of the baseline transurethral resection (TUR) samples. Patients were excluded from the current analysis in the following cases: sequenced samples without clinical information or patients with clinical informations without sequencing results; uncertain sequencing outcomes (due to technical reasons); neuroendocrine histology; immunotherapy or anti-FGFR therapy after progression (to avoid a potential influence on survival data).

The main clinical and demographic data included gender, age, stage and previous therapies. The surgical specimen was graded according to WHO classification and staged by the 7th TNM criteria. The patients’ basic pathological (histology, pT, pN, demography, age, gender), clinical, oncological treatment and outcome data were collected from the pathological and medical documents of University of Szeged, and the overall survival data from the National Health Insurance Fund database, respectively. All data of patients from different databases were linked at the patient level then de-identified. Overall survival (OS) was defined from the date of cystectomy to the date of death.

### Tissue sample testing

Two tests were performed on easch tissue sample. The service provider together with University of Szeged performed FGFR next-generation sequencing (NGS) for mutations and PD-L1 stain with DAKO 28-8 tests.

This sample collection was supplemented with a retrospectively analyzed anonymized patient’s follow up database from the medical reports and funder data.

Only the FGFR3 mutation status (wild type -WT, non-wild type—NWT) was recorded, the exact type of mutation (point mutation, deletion, insertion, etc.) was not analyzed. The PD-L1 (IHC) expression level of the samples was given in percentages, and the samples were considered positive if the expression level was at least 1% and negative otherwise.

PD-L1 positivity was defined if the PD-L1 expressed tumor cell count was at least 1% (tumor positive score—TPS). Nowadays, a more relevant CPS score in clinical application has also been defined as the ratio of the number of all PD-L1–expressing cells (tumor cells, lymphocytes, macrophages) to the number of all tumor cells (high level ≥10) (9).

A detailed description of histological and molecular analyses can be found in the Supplementary Material.

### Formation of analyzed groups

**FIGURE 1:**
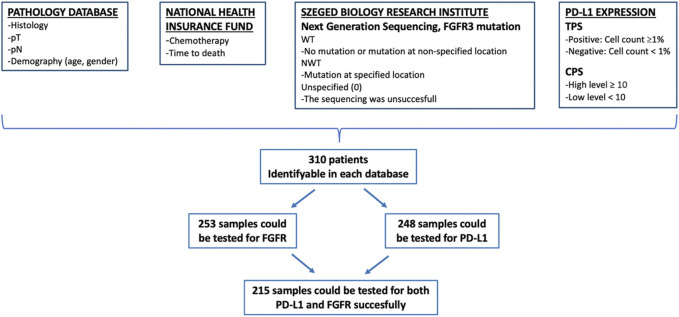
Method of data collection. CPS, combined positive score; FGFR, fibroblast growth factor receptor; NWT, non wild type; PD-L1, programmed cell death ligand 1; pN, pathologic lymph node stage; pT, pathologic tumor stage; TPS, tumor positive score; WT, wild type.

In our study 392 surgical samples were collected, of which 82 patients were excluded on the basis of insufficient information. The data of 310 patients were considered for analysis. Three subgroups were formed based on possible testing for FGFR, PD-L1 and CPS score: in the first subgroup of patients, FGFR mutation testing of histological samples were performed; in the second subgroup, PD-L1 analysis was available; while in the third subgroup, both tests (PD-L1 and FGFR) were also performed. The data on the interaction of biomarkers and their role in survival were evaluated in the last subgroup in which all results were available (Figure 1).

### Statistical analysis

Demographic data were characterized using gender, median age, TNM stage and different biomarkers. The independence between the stratifying variables was analyzed using chi-square test for independence. Fischer’s Exact test was used to examine the relationship between binary variables. P values <0.05 were considered significant.

Survival analysis was performed to analyze overall survival, Kaplan-Meier estimators were used to characterize the survival function. The effect of TNM, FGFR mutation, and PD-L1 expression on overall survival (OS) was evaluated independently using univariate stratification of the Kaplan-Meier estimation. During survival analyses, Bonferroni correction was used for pairwise comparisons in the case of variables with more than 2 groups. Univariate and multivariate (with forward likelihood ratio method) Cox proportional hazard models were used to estimate the effect of certain covariates on the overal survival from cystectomy. The following variables were used in the Cox models as predictors: gender, age of the patient at the time of cystectomy (dichotomized as under 65 years vs. at least 65 years), TNM stage, FGFR mutation (WT/NWT), PD-L1 expression (positive/negative), chemotherapy (yes/no). Model reference values were the following: gender–male, age–lower than 65 years, chemotherapy–no, TNM stage–pT0_cyst_ pN0, FGFR–NWT, PD-L1—negative.

SPSS 25.0 for Windows (SPSS Inc., Chicago, IL, United States) was used for statistical analysis. Survival analyses (Kaplan-Meier plots) were carried out using the statistical software R 4.2.2 (R Core Team 2021).

## Results

### Baseline characteristics, TNM stage, FGFR and PD-L1 results

The data of 310 patients were considered for analysis, of 236 (76.1%) were male and 74 (23.9%) female. The median age of the entire patient population was 62.8 years, women were slightly younger (median age 61.5 years) than men (median age 63.1 years) (Table 1). 253 samples could be tested for FGFR mutation, 248 samples for PD-L1 and CPS score, and 215 samples for both PD-L1 and FGFR succesfully. The characteristics of the patients were similar in the entire population, as in the further analyzed subgroup in which both biomarkers could be evaluated (Figure 1 and Table 1).

**TABLE 1:** The baseline characteristics.

	All patients (%) [valid %]	Both FGFR and PD-L1 available (%)
Number	310 (100)	215 (69.4 of all pts)
Gender		
Male	236 (76.1)	171 (79.5)
Female	74 (23.9)	44 (20.5)
Age		
Median age (months)	62.8	62.9
Patients over 65 years (%)	112 (36.1)	80 (37.2)
Stage (%)		
pT0_cyst_ pN0	20 (6.5)	19 (8.8)
St.0-I (pTa, pTis, pT1 pN0)	54 (17.4)	43 (20.0)
St.II (pT2a, pT2b pN0)	60 (19.3)	41 (19.1)
St.III-IV (pT3a, pT3b, pT4/pN+)	176 (56.8)	112 (52.1)
Any chemotherapy performed (%)	87 (28.1)	59 (27.4)
Neoadjuvant (NA) chemotherapy (%)	18 (5.8)	12 (5.6)
Any chemotherapy except NA (%)	69 (22.3)	47 (21.8)
FGFR		
Missing or unsuccesful	95 (30.7)	NA
Non-wilde type (NWT)	36 (11.6) [16.7]	36 (16.7)
Wilde type (WT)	179 (57.7) [83.3]	179 (83.3)
PD-L1 (TPS)		
Missing or unsuccesful	62 (20.0)	NA
PD-L1 negative (<1%)	146 (47.1) [58.9]	129 (60.0)
PD-L1 positive (≥1%)	102 (32.9) [41.1]	86 (40.0)
PD-L1 (CPS)		
Missing or unsuccesful	62 (20.0)	NA
CPS < 10	169 (54.5) [68.1]	146 (67.9)
CPS ≥ 10	79 (25.5) [31.9]	69 (32.1)

CPS, combined positive score; FGFR, fibroblast growth factor receptor; NA, not applicable; NWT, non-wilde type; PD-L1, programmed cell death ligand 1; pts, patients; St, stage; TPS, tumor positive score; WT, wilde type.

Results of FGFR alteration testing were categorized into subgroups based on the non-wild type or wild type, PD-L1 immunostaining data as TPS negative or positive, and CPS <10 or CPS ≥10, respectively (Table 1).

### Test of independence of TNM stage, FGFR and PD-L1 status

There was a strong correlation between TNM stage and FGFR mutation (*p* < 0.001), i.e., higher stage had a lower NWT ratio. The positive PD-L1 rate was significantly (*p* = 0.005) lower in the NWT group (19.4% vs. 44.1%) than in the WT, similar to the CPS ≥10 rate (*p* = 0.003). Significant relationship was also found between stage and PD-L1 expression based on TPS (*p* = 0.070) or CPS (*p* = 0.002), in more advanced stages the frequency of PD-L1 positivity was higher (Tables 2–4).

**TABLE 2:** Correlations between the FGFR mutation and the TNM stage.

		FGFR alteration		*p*-value
		NWT	WT	
		*n* = 36	*n* = 179	
TNM stage	pT0_cyst_ (%)	5 (26.3)	14 (73.7)	**<0.001**
	St.I (%)	16 (37.2)	27 (62.8)	
	St.II (%)	7 (17.1)	34 (82.9)	
	St.III-IV (%)	8 (7.1)	104 (92.9)	

**TABLE 3:** Correlations between the FGFR mutation and the PD-L1 expression (TPS, CPS).

		FGFR alteration		*p*-value
		NWT	WT	
		*n* = 36 (%)	*n* = 179 (%)	
PD-L1 expression (TPS)	Negative (<1%)	29 (80.6)	100 (55.9)	**0.005**
	Positive (≥1%)	7 (19.4)	79 (44.1)	
PD-L1 expression (CPS)	<10	32 (88.9)	114 (63.7)	**0.003**
	≥10	4 (11.1)	65 (36.3)	

**TABLE 4:** Correlations between the TNM stage and the PD-L1 expression (TPS, CPS).

		PD-L1 expression					
		TPS < 1%	TPS ≥ 1%	*p*-value	CPS < 10	CPS ≥ 10	*p*-value
		*n* = 129	*n* = 86		*n* = 146	*n* = 69	
TNM stage	pT0_cyst_	12 (63.2)	7 (36.8)	**0.07**	14 (73.7)	5 (26.3)	**0.002**
	St.I	33 (76.7)	10 (23.3)		39 (90.7)	4 (9.3)	
	St.II	24 (58.5)	17 (41.5)		23 (56.1)	18 (43.9)	
	St.III-IV	60 (53.6)	52 (46.4)		70 (62.5)	42 (37.5)	

We focused primarily on the correlation between FGFR and PD-L1 (TPS and CPS) status, where we found that the more likely the samples were FGFR mutated, the less likely they were PD-L1 positive. Our results show that TNM stage has a strong significant effect on FGFR mutation and PD-L1 expression.

### Effect of TNM stage on survival

**FIGURE 2:**
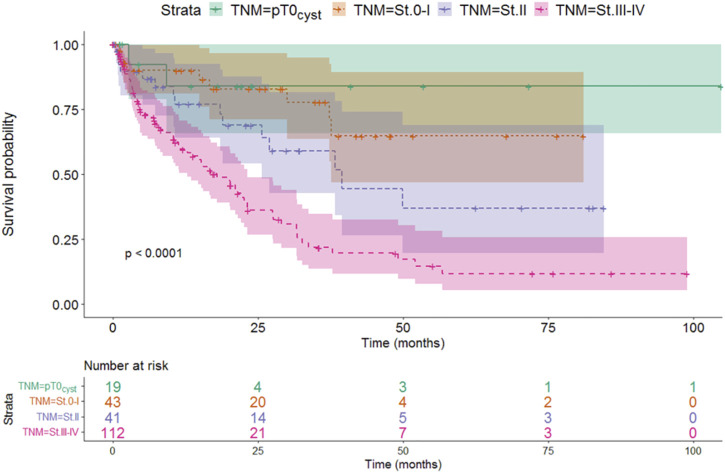
The effect of TNM stages on survival.

Stratifying the patients based on the TNM stage at the time of cystectomy showed that the survival at more advanced stages was worse than at earlier cases. Survival of locally advanced patients with TNM stage III-IV at the time of cystectomy was significantly the most unfavorable factor (median: 17.97 months, *p* < 0.001). Although this is not unexpected, this finding verifies the validity of the model (Figure 2 and Table 5).

**TABLE 5:** Connection with TNM stages and median survival.

TNM stage	Median survival (months)	95% CI LL	95% CI UL	*p*-value
pT0_cyst_	Not reached	NA	NA	<0.001
St.0-I	Not reached	37.86	NA	
St.II	39.50	20.25	58.75	
St.III-IV	17.97	11.98	23.96	

CI, confidence interval; LL, lower limit; St, stage; TNM, Tumor, Node, Metastasis; UL, upper limit.

Based on the pairwise comparison, we found that the survival of the TNM stage III-IV group was significantly worse compared to the other groups, while no difference could be detected between the groups with a better prognosis.

### The effects of analyzed biomarkers on survival

**FIGURE 3:**
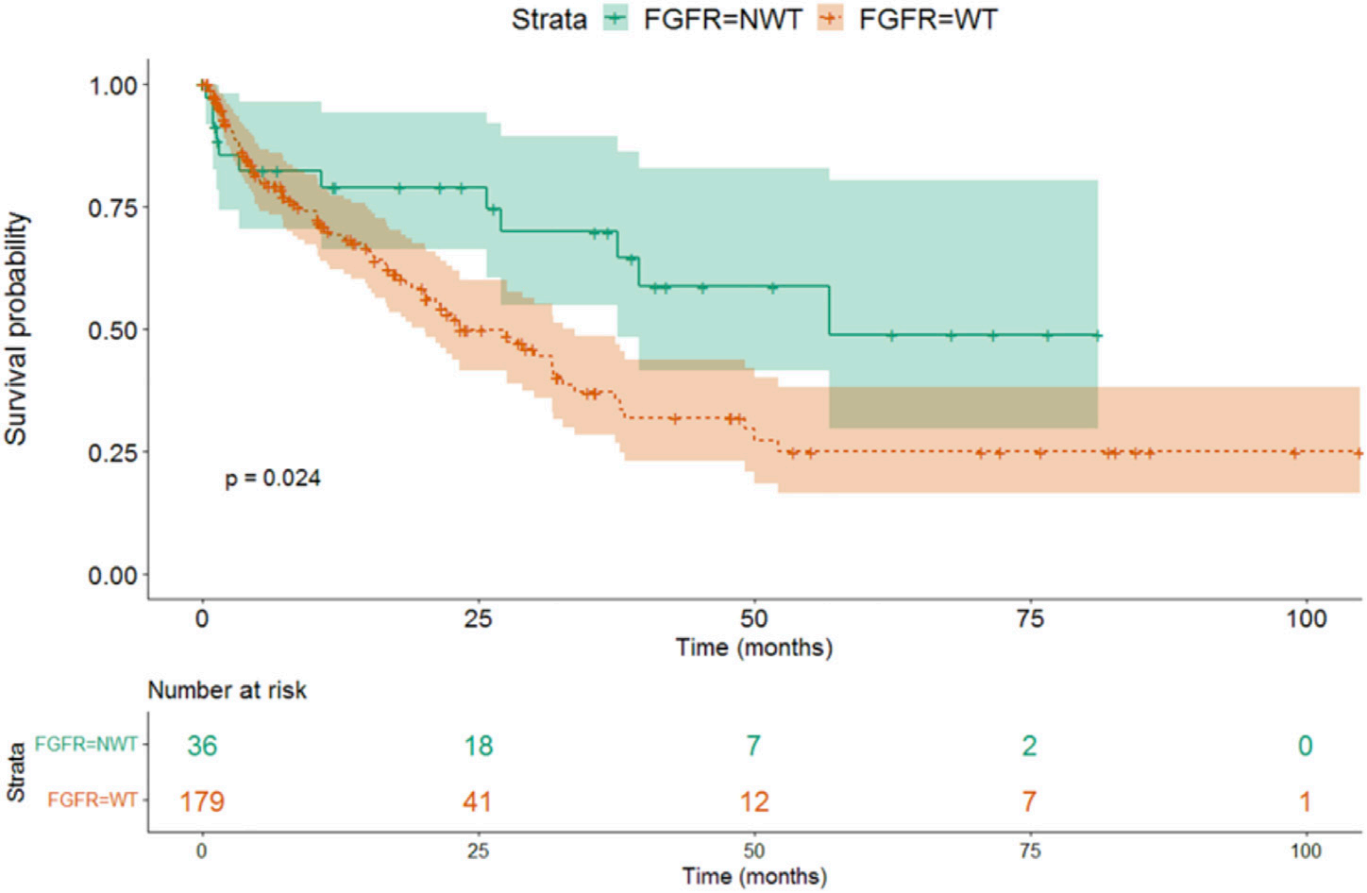
The effect of different FGFR mutations on survival.

We found that the survival was longer in FGFR positive, mutant (NWT - median OS 56.7 months, 95% CI 38.9-NA), than in FGFR wild type (WT—median OS 23.2 months, 95% CI 15.6–30.9) patients (*p* = 0.024) (Figure 3 and Table 6).

**TABLE 6:** Connection with FGFR mutations and median survival.

FGFR	Median survival (months)	95% CI LL	95% CI UL	*p*-value
NWT	56.73	38.95	NA	0.024
WT	23.23	15.59	30.87	

CI, confidence interval; FGFR, fibroblast growth factor receptor; LL, lower limit; NA, not available; NWT, non-wild type; WT, wild type; UL, upper limit.

**FIGURE 4:**
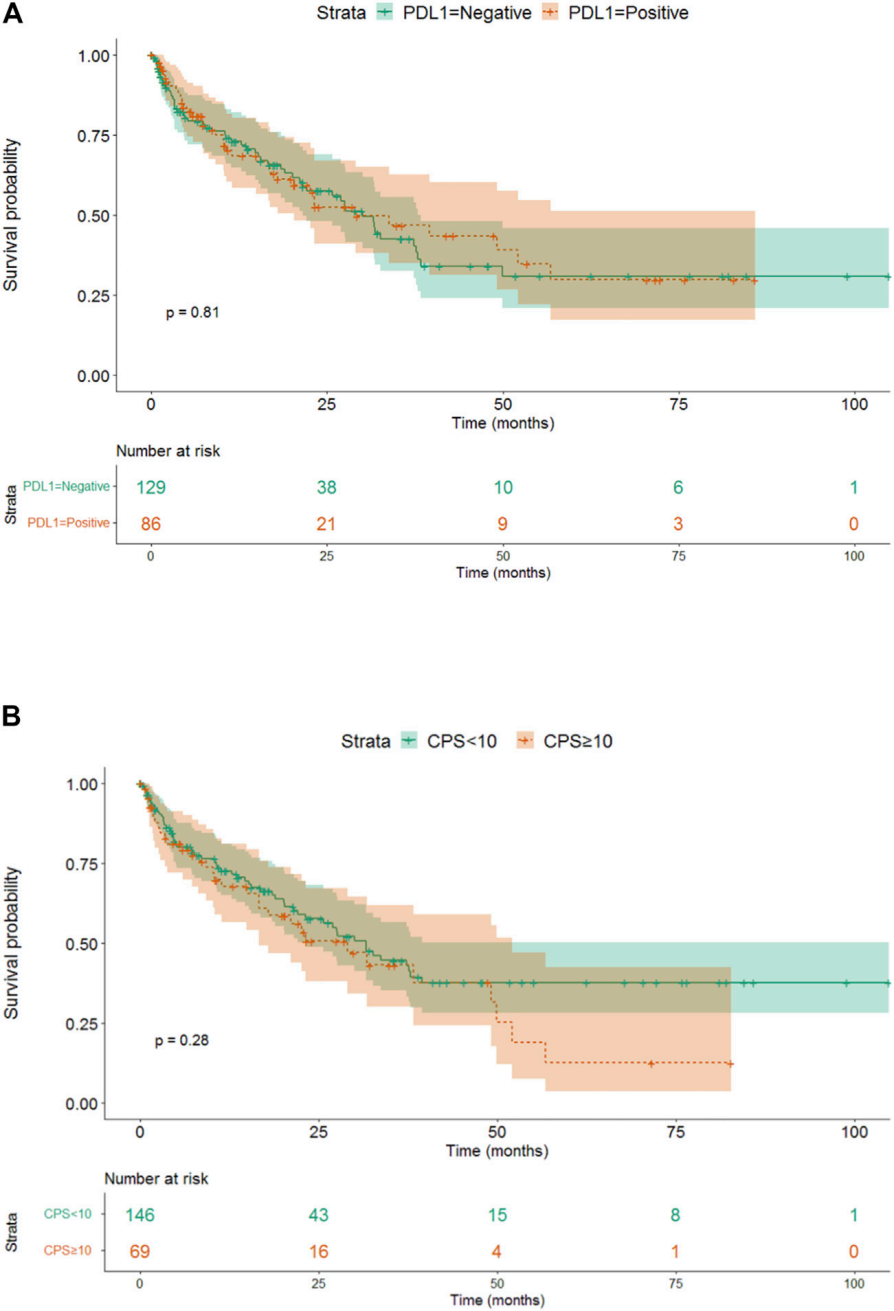
**(A)** The effect of different 256 PD-L1 expressions (TPS) on survival. **(B)** The effect of different PD-L1 expressions (CPS) on survival.

There was no difference detected in median overall survival between patients with PD-L1 positive or negative (30.07 vs. 29.03, *p* = 0.81) based on TPS, and high or low level of CPS (31.63 vs. 29.03, *p* = 0.28) (Figures 4A, B).

Based on our data, FGFR NWT vs. WT was a factor affecting patient survival, while PD-L1 negativity vs. positivity or CPS low vs. high level was not found significant. Our data showed that the stage proved to be a significant independent factor for survival, the close connection with FGFR had no independent effect. As in case of TNM, the independency of these variables were rejected with high probability here too.

The gender (male vs. female, HR: 1.18, *p* = 0.52), age (older than 65 vs. younger, HR: 1.48, *p* = 0.07) of the patients and the chemotherapy use (no versus yes, HR: 0.793, *p* = 0.18) did not affect survival.

There was a significant correlation between all variables (TNM, FGFR status, PD-L1 status). All covariates were associated with TNM stage and their impact on survival is through the TNM stage. It is not possible to evaluate the impact of any of the covariates independently from each other, based on multivariate Cox model the only exception is TNM stage.

Hazard ratios for the covariates in univariate and multivariate analysis from Cox model are summed in Table 7.

**TABLE 7:** Hazard ratios for the covariates in univariate and multivariate analysis.

Covariate	Univariate				Multivariate			
	HR	95% CI LL	95% CI UL	*p*-value	HR	95% CI LL	95% CI UL	*p*-value
TNM stage								
pT0_cyst_	References							
St.0-I	1.612	0.348	7.469	0.541	1.612	0.348	7.469	0.541
St.II	3.156	0.721	13.803	0.127	3.156	0.721	13.803	0.127
St.III-IV	6.812	1.665	27.857	0.008	6.812	1.665	27.857	0.008
FGFR								
NWT	References							
WT	1.997	1.082	3.685	0.027	NA			0.718
PD-L1								
TPS < 1%	References							
TPS ≥ 1%	1.052	0.693	1.596	0.813	NA			0.231
CPS < 10	References							
CPS ≥ 10	1.265	0.825	1.938	0.281	NA			0.776

CI, confidence interval; CPS, combined positive score; FGFR, fibroblast growth factor receptor; HR, hazard ratio; LL, lower limit; NA, not available; NWT, non-wild type; PD-L1, programmed cell death ligand 1; St, stage; UL, upper limit; WT, wild type.

## Discussion

It is well-known that urothelial tumors of the bladder predominantly develop in older adults, owing to the influence of environmental factors. However, such tumors have begun to appear more and more often in younger age groups (19). In our analysis, the average age of patients was 63 years; 36% were over 65 years old, and women were slightly younger than men.

The standard treatment of muscle-invasive, non-metastatic tumors is cystectomy. Surgery may or may not be preceded by neoadjuvant chemotherapy, depending on eligibility to receive cisplatin (1, 3). Neoadjuvant cisplatin-based chemotherapy results in a 5% benefit in 5-year overall survival and a 9% benefit in 5-year disease-free survival based on a meta-analysis (11 trials, 3,005 patients) (20)**.** Most of our analyzed cases were locally advanced, but the use of chemotherapy was still detected at a very low rate. This may be due to the fact that in the period covered, the only treatment option available was the toxic platinum-based chemotherapy combination, which generally yielded little success. As a result, it was used with great caution and viewed with skepticism by both patients and urologists (21). In our study, the use of chemotherapy did not provide a survival advantage in the entire population. Since the focus of our work was the investigation of biomarkers and the number of patients receiving chemotherapy was small, it was not possible to search for further relevant correlations related to chemotherapy.

In recent years, the appearance of immunotherapy has been a breakthrough in the treatment of UBC, nowadays clinical trials are also taking place with the use of neoadjuvant indications (22). Radical surgery can also be recommended in the case of multiple recurring, non-muscle invasive tumors, as was the case in 18.7% of our results, if the patient’s general condition allowed it (1, 3).

The indication for cystectomy is mainly the muscle invasive diagnosis based on TUR. However, it is possible that tumor tissue is no longer detectable in the cystectomy sample (pT0), e.g., due to the ablative effect of TUR or even the effectiveness of neoadjuvant treatment. The life expectancy of these patients is more favorable based on literature data (23). pT0 tumor status occurred in 6.5% of our samples, the survival of these patients was the best amongst all stages, similar to the international multicenter results of Tilki et al (23).

PD-L1 expression and FGFR alterations are the most frequently investigated biomarkers in connection with the treatment of advanced bladder tumors today, due to the clinical need related to therapeutic options. Immunotherapies are mostly used in advanced urothelial cancer after platinum-based chemotherapy, regardless of biomarker analysis, but they are also effective and approved as a first choice in case of high PD-L1 (CPS–combined positive score or IC–immune cell score) status (1, 3). In our work, PD-L1 status was determined based on TPS and CPS as well. Their effect on the outcome of the disease had a similar prognostic value. PD-L1 expression on urothelial tumor cells was associated with muscle-invasive disease and with worse overall survival (24).

TCGA project supports the high molecular heterogenity of MIBC such as in non-small cell lung cancer and in melanoma (25). The most common form of bladder cancer is NMIBC at diagnosis. Histologically these tumors are papillary tumors, they recur in more than the half of the cases, but have rare progression tendency (26). Approximately 70% of low-grade non-invasive papillary tumors show FGFR3 mutation in literature (27). In our study, patients with superficial bladder tumors (20%) who underwent cystectomy were included after a recurrence or if the disease could not be controlled by transurethral resection. Even in this higher-risk superficial group, the proportion of FGFR mutant patients was 37.2%, lower than in published data, but higher than in our analyzed muscle-invasive or locally advanced group (30). The strongest correlation could be observed between TNM stage and FGFR mutation. Our results represent the high frequency of FGFR3 mutation in earlier stages. Previous studies support our data, and it has been demonstrated that over half of pTa tumors recur, accordingly FGFR alteration is a possible signaling pathway in the development of these tumors (27). Compared to the literature (26), our findings suggest an oncogenic relationship, as FGFR3 mutation is mostly seen in non-invasive tumors, and less frequently at more advanced stages. Fernandez et al. found FGFR genomic alterations as an independent factor associated with the survival and as a relevant biomarker of mUC that may influence response to systemic therapy (28).

Advanced stage tumors have worse survival than early stage, and stratifying the patients according to TNM stage, we found also significant difference in survival from cystectomy between NWT and WT patients. Our data is similar to other published results that have reported an association between favorable prognosis and FGFR mutation status (29). The real prognostic effect of FGFR mutation is questionable because of the strong correlation with low stage tumors. Based on our results, FGFR alteration is not an independent prognostic parameter for survival, but occurs more often at lower stages, which is why it affects the overall survival of patients through the stage.

We detected across TNM stages that tumors with high ratio of FGFR3 mutation are less likely associated with positive PD-L1 expression. Regarding the covariates examined with the cox regression model, we found that their occurrence is not independent of each other. Based on the results obtained, a very strong correlation could be identified between the individual parameters, but the database analysis method and the limited number of elements found in each subgroup did not allow the matching of the individual elements and the adjustment of the data.

Even if there is an assumable connection between these results, there is no clear evidence that FGFR3 alteration would enhance a resistance mechanism against immune checkpoint inhibitors. Some previous studies verified mutated FGFR3 with increased FGFR3 gene expression and an association with decreased T-cell infiltration, but in this publication there was no significant difference in response rate or OS with immunecheckpoint inhibitors in FGFR3 separated groups, possibly due to the lower stromal-mediated immune suppression (17). The controversial manifestation of FGFR3 and PD-L1 in various stages of the examined cystectomic samples in our study suggest a deeper stratification in molecular and immunological status in urothelial carcinomas.

The T-cell based subtyping of bladder cancers shows that tumors with high FGFR3 expression are associated with lower T-cell infiltration based on the count of the CD8^+^ T-cells (16). These findings may propose a negative or immunosuppressive effect of FGFR3 alterations on T-cell gene mechanism. Based on one of the latest retrospective analyses with a relatively high number of patients available in the literature, a lower response rates and shorter OS was observed in patients with FGFR alteratations following anti-PD-L1 immunotherapy (18).

Our aim was to investigate whether FGFR mutation is a possible independent prognostic factor of survival. Reflect on many controversial survival and response data in the anti-PD-L1 treated FGFR mutated patient group (15, 18, 30–33), according to other studies we consider a larger investigation of special non-invasive subtypes to be necessary in order to verify its predictive and prognostic value. In addition, we consider it forward-looking waiting for the results of the phase 3 prospective THOR (NCT03390504) study, which compares the effects of erdafitinib and pembrolizumab in patients with advanced mUC, to clarify the real therapeutic significance of FGFR alterations (34).

Limitations of our study include that we could not obtain retrospective relevant clinical data in almost 1/3 of the cystectomized patients, and molecular analysis was unsuccessful in 15% of the samples suitable for FGFR analysis. Another limitation is that although a strong correlation was detected between the individual investigated parameters, due to the limited number of elements of each subgroup, and the type of database analysis method, matching the individual elements and the adjustment of the data was not feasible.

The strength of our work is that it processes the real-life results of a relatively large number of bladder tumor patients who have undergone cystectomy (35). Another advantage of our work is that we also evaluated the CPS data in relation to PD-L1 expression, used better in the daily practice during first line immunotherapies nowadays, which would provide the opportunity for further potentially predictive conclusions. It should also be emphasized that it was possible to connect the data available in the clinical and pathological medical systems precisely and individually with the survival results available in the funder’s database, thus facilitating the accuracy of our work.

Our results highlight the high FGFR alteration rate in non-muscle invasive tumors, thereby pointing to a potentially new area for future analysis of the effect of FGFR inhibitors. The higher rate of PD-L1 expression in more advanced stages also confirms the immune mechanism of bladder tumors. Although the survival of FGFR mutant patients was more favorable than wild-type, this effect was established through the tumor stage.

In summary, based on all of our results, the role of tumor stage can be highlighted as the strongest survival factor in this group of patients.

While molecular subtyping of urothelial cancers has yet to find its exact place in managing the disease, more and more data are being collected on the molecular profile of each subtype. The goal of newer clinical trials is to combine immunotherapy with modern, antigen-drug conjugates, and to find a place for targeted therapies against individual genetic abnormalities. Although immunotherapy is now the standard treatment for UBC, the frequency of FGFR3 alterations in NMIBC underscores the importance of a new molecular classification for the future of targeted therapy. FGFR inhibitors may represent an additional solution in the treatment of urothelial cancer, perhaps in a possible combination of immune and molecularly targeted therapies, or in halting the progression of early-stage FGFR-mutant tumors.

## Data Availability

The raw data supporting the conclusion of this article will be made available by the authors, without undue reservation.

## Abbreviations

CD8, cluster of differentiation 8; CPS, combined positive score; FFPE, formalin-fixed paraffin-embedded; FGFR, fibroblast growth factor receptor; HR, hazard ratio; IHC, immunohistochemistry; LCL, lower control limit; MIBC, muscle-invasive; NGS, next-generation sequencing; NMIBC, non-invasive; NWT, no wild type; OS, overall survival; PCR, polymerase chain reaction; PD-1, programmed cell death-1; PD-L1, programmed cell death-ligand 1; St, stage; TCGA, the cancer genom atlas; TNM, tumour, node, metastasis; TPS, tumor positive score; TUR, transurethral resection; UBC, urothelial bladder cancer; UCL, upper control limit; WT, wild type.
