# Supplementary material for: Correlation between fibroblast growth factor receptor mutation, programmed death ligand-1 expression and survival in urinary bladder cancer based on real-world data
Source: Pathol Oncol Res. 2023 Apr 21;29:1611077. doi: 10.3389/pore.2023.1611077 (PMC10160374; doi:10.3389/pore.2023.1611077)
Supplement: Supplementary file 1 [file DataSheet1.doc]

**SUPPLEMENTARY MATERIALS**

Supplementary data of histological and molecular analyses

The samples were anonymized according to the Bio Bank Regulation (250/C-2/2017K.K) of the University of Szeged. The anonymized formalin-fixed paraffin-embedded (FFPE) samples, coded with numbers, were provided to the DeltaBio 2000 Ltd. by the Department of Pathology (University of Szeged). Samples were examined in the laboratories located at the Pathology Department and at the Biological Research Centre of Szeged similarly to the daily molecular pathology diagnostic work. The laboratory work including immunohistochemistry (IHC) and Polymerase Chain Reaction (PCR)/sequencing were organized by DeltaBio 2000 Ltd.

Immunostaining was performed on the tissue samples for the testing for programmed death ligand-1 (PD-L1) expression. Baseline PD-L1 expression was evaulated using 28-8 pharmDX (DAKO) immunoassay. The PD-L1 IHC reaction with 28-8 pharmDX (DAKO) was performed at the University of Szeged based on a research and development contract with Janssen.

Immunostaining analysis was characterized by the quality level of the result. This quality level can range from 1 to 4. When the result had a quality level of 1 or 2, the result was considered less reliable, therefore these results were not taken into consideration.

The anonymized FFPE samples for the NGS analyses, coded with numbers, were provided by the University of Szeged to the Delta Bio 2000 Ltd. for deoxyribonucleic acid (DNA) / ribonucleic acid (RNA) isolation and PCR/sequencing. PCR/sequencing was directed and quality assured by DeltaBio 2000 Ltd. Macrodissected FFPE samples are provided by the Pathological Institute. DNA isolation was carried out using the MagCore® Genomic DNA FFPE One-Step Kit with the high yield protocol. DNA samples were processed according to the manufacturer’s recommendations. If the spectrophotometrically determined quality of the isolated DNA was to be insufficient for downstream application, one repeated DNA isolation was performed. Genomic DNA with more than 10 ng/µl is subjected to further mutation analysis. The presence of mutations was analyzed by multiplex PCR-based targeted NGS sequencing. The general workflow for sample preparation includes a two-step PCR procedure to amplify 3 regions in the fibroblast growth factor receptor (FGFR) 3 gene: exon 7, 10, and 15 followed by a nested PCR with the barcoded primers, included sequencing adapters. Gene-specific primers were designed to amplify a region of 164 bp in the FGFR3 exon 7 (genomic coordinates after GRCh38 are: chr4:1,801,778-1,801,941), a region of 192 bp in the FGFR3 exon 9 (genomic coordinates after GRCh38 are: chr4:1,804,320-1,804,511), and a region of 152 bp in the FGFR3 exon 15 (genomic coordinates after GRCh38 are: chr4:1,806,101-1,806,252). PCR amplicons were verified by agarose gel electrophoresis. In case of missing PCR fragments, repeated amplification is performed with the appropriate primer pairs. If the repeated amplification does not result in any product, the sample is excluded from the following NGS library preparation step.The barcoded PCR products were quantified by qPCR. The pooled amplicons were then mixed 9:1 with PhiX (Illumina), denatured, and clustered at 1 pM on a Miniseq flow-cell and sequenced. Raw files were processed in paired-end mode to remove adapter sequences and to filter out pairs with a sequence < 100 nt to exclude short read artefacts. Local alignments of reads to the hg38 genome, and position-specific nucleotide metrics within the co-ordinates of the amplified loci are performed with custom-written script. Mutations were called where a non-reference base was presented at a frequency of between 2.5–97.5% in both directions. The Service provider provide the results in annotated files.

On some tissue samples the FGFR testing could not be performed due to tissue age and other inadequate qualities, therefore consider: in these cases the FGFR status could not be determined.
